# The Physical Activity at Work (PAW) Program in Thai Office Workers: Mixed Methods Process Evaluation Study

**DOI:** 10.2196/57604

**Published:** 2025-01-02

**Authors:** Katika Akksilp, Thomas Rouyard, Wanrudee Isaranuwatchai, Ryota Nakamura, Falk Müller-Riemenschneider, Yot Teerawattananon, Cynthia Chen

**Affiliations:** 1 Health Intervention and Technology Assessment Programme Ministry of Public Health Nonthaburi Thailand; 2 Saw Swee Hock School of Public Health National University of Singapore and National University Health System Singapore Singapore; 3 Graduate School of Public Health & Health Policy City University of New York New York, NY United States; 4 Hitotsubashi Institute for Advanced Study Hitotsubashi University Tokyo Japan; 5 Yong Loo Lin School of Medicine National University of Singapore and National University Health System Singapore Singapore; 6 Digital Health Center Berlin Institute of Health Charité-Universitätsmedizin Berlin Berlin Germany

**Keywords:** process evaluation, sedentary behavior, physical activity, workplace, movement breaks

## Abstract

**Background:**

An increasing number of multicomponent workplace interventions are being developed to reduce sedentary time and promote physical activity among office workers. The Physical Activity at Work (PAW) trial was one of these interventions, but it yielded an inconclusive effect on sedentary time after 6 months, with a low uptake of movement breaks, the main intervention component.

**Objective:**

This study investigates the factors contributing to the outcomes of the PAW cluster randomized trial.

**Methods:**

Following the Medical Research Council’s guidance for process evaluation of complex interventions, we used a mixed methods study design to evaluate the PAW study’s recruitment and context (how job nature and cluster recruitment affected movement break participation), implementation (dose and fidelity), and mechanisms of impact (assessing how intervention components affected movement break participation and identifying the facilitators and barriers to participation in the movement breaks). Data from accelerometers, pedometers, questionnaires, on-site monitoring, and focus group discussions were used for the evaluation. Linear mixed effects models were used to analyze the effects of different intervention components on the movement breaks. Subsequently, qualitative analysis of the focus group discussions provided additional insights into the relationship between the intervention components.

**Results:**

The participation in movement breaks declined after the third week, averaging 12.7 sessions (SD 4.94) per participant per week for the first 3 weeks, and continuing to decrease throughout the intervention. On-site monitoring confirmed high implementation fidelity. Analysis of Fitbit data revealed that each additional movement break was associated with a reduction of 6.20 (95% CI 6.99-5.41) minutes in sedentary time and an increase of 245 (95% CI 222-267) steps. Regarding the mechanisms of impact, clusters with higher baseline sedentary time demonstrated greater participation in movement breaks, while those with frequent out-of-office duties showed minimal engagement. Moreover, clusters with enthusiastic and encouraging movement break leaders were associated with a 24.1% (95% CI 8.88%-39.4%) increase in participation. Environmental and organizational support components using posters and leaders’ messages were ineffective, showing no significant change in percentage participation in movement breaks (4.49%, 95% CI –0.49% to 9.47% and 1.82%, 95% CI –2.25% to 5.9%, respectively). Barriers such as high workloads and meetings further hindered participation, while the facilitators included participants’ motivation to feel active and the perceived health benefits from movement breaks.

**Conclusions:**

Despite high fidelity, the PAW trial did not significantly reduce sedentary time, with limited uptake of movement breaks due to context-related challenges, ineffective environmental support, and high workloads during the COVID-19 pandemic.

## Introduction

### Background

Extensive research has examined workplace interventions to reduce sedentary behavior and promote physical activity globally [1,2]. Numerous interventions have yielded noteworthy results, showcasing their effectiveness, whereas some have encountered challenges in achieving desired outcomes [2-5]. While early meta-analyses, such as one from 1998, suggested minimal impact of workplace interventions on physical activity [6], subsequent reviews, including one from 2003, began to recognize their potential benefits [7]. However, more recent evidence has provided a clearer picture, indicating that more successful interventions tended to use pedometers, apply internet-based approaches, and include activities at social and environmental levels [5]. One systematic review found that physical environment interventions, whether implemented at the workstation, building, or neighborhood level, were effective in promoting physical activity in the workplace [8]. Finally, complex or multicomponent interventions emerge as the most effective workplace strategy for reducing sitting time [4], highlighting the intricate process leading to successful behavioral changes [9].

However, multicomponent intervention trial reports often fail to elucidate the rationale and mechanisms behind their results, leaving readers with questions regarding the efficacy of the interventions. Many scholars have criticized these trials as being akin to a “black box” because the underlying reasons for their success or failure remain unknown [10]. As a result, process evaluations, which assess how interventions were implemented in practice, are essential for deciphering the implications of the results from multicomponent intervention trials. These evaluations highlight aspects of the intervention that may require improvement to increase the probability of success [11,12].

Several frameworks advocate for process evaluations to explore the context, implementation, and impact mechanisms of intervention programs, encompassing aspects such as recruitment, reach, dose, fidelity, and challenges [10-13]. Process evaluations have become particularly relevant because they can help determine whether success or failure lies in the implementation, the intervention itself, or a combination of both factors. Notably, the Medical Research Council’s guidance on process evaluation of complex interventions is particularly valuable for assessing multicomponent interventions [11]. By contrast, frameworks like the one proposed by Steckler and Linnan [13] focus more on specific elements such as dose and fidelity but may not provide the broader perspective necessary to understand the interactions between context, mechanisms, and implementation. The emphasis of the Medical Research Council’s guidance on context and mechanisms was pivotal in capturing the socioecological dynamics within our intervention.

In addition, there is increasing acknowledgment that incorporating both qualitative and quantitative data, as well as using theoretical frameworks within process evaluations, plays a crucial role in promoting evidence-based practice [14]; for example, the Older People’s Exercise Intervention in Residential and Nursing Accommodation trial [15] used a mixed methods process evaluation to assess whether the multicomponent intervention changed residents’ home culture to increase physical activity and whether residents engaged in exercise activities. The study found no notable cultural shifts or sufficient engagement, thus explaining the trial’s null results [15,16]. Another process evaluation explored the implementation and impact mechanisms of a park prescription intervention trial, revealing key mediators of the intervention effects, such as park physical activity levels, as well as barriers that may have weakened intervention effectiveness [17].

### Objectives

In Thailand, it has been reported that the majority of adults who engage in sufficient physical activity reside in rural areas, typically due to their work in the agricultural sector, whereas physical activity levels tend to be lower among office workers [18]. Numerous nonresearch initiatives have been launched to promote physical activity and reduce sedentary behavior among office workers in Thai companies and organizations. The Physical Activity at Work (PAW) trial [19] marked the first comprehensive cluster randomized trial aimed at promoting physical activity and reducing sedentary behavior among Thai office workers. This trial incorporated a multicomponent intervention and used accelerometer-measured data, laying a solid foundation for physical activity research in Thailand [20]. However, the trial produced inconclusive findings, leaving critical questions regarding the factors contributing to its outcomes unanswered. We observed a suboptimal uptake of movement breaks, the primary intervention component, and inferred that this, coupled with a low recruitment rate, may explain the absence of statistically significant outcomes [20]. Understanding these underlying reasons is essential for making necessary improvements in both research and policy. To address this, we conducted a process evaluation to investigate the factors contributing to the trial’s outcome.

Following the Medical Research Council’s guidance for process evaluation of complex interventions [11], we used a mixed methods study design to evaluate the PAW study’s recruitment and context, implementation, and impact mechanisms. Specifically, we examined how job nature and recruitment processes within each cluster influenced participation in movement breaks (context), the overall dose and fidelity of the intervention (implementation), and the effects of intervention components on participation in movement breaks as well as the facilitators and barriers to movement break participation (impact mechanisms).

## Methods

### Description of the Cluster Randomized Trial

The PAW study was conducted in a free-living setting at the Ministry of Public Health offices in Nonthaburi, Thailand. Detailed methodological information regarding the cluster randomized trial (Thai Clinical Trials Registry: TCTR20200604007) was published in the protocol [19] and main results [20] manuscripts. Briefly, between July and September 2021, we recruited 282 office workers (age: mean 38.6, SD 10.4, years; female: n=228, 80.9%) from 18 separate offices. After baseline data collection, the participants were randomized into 9 intervention offices (140/282, 49.6%) and 9 control offices (142/282, 50.4%). The 6-month intervention, which took place between September 2021 and March 2022, consisted of 6 components across 4 levels, as outlined in Textbox 1.

**Key elements of the Physical Activity at Work cluster randomized trial.**

**Individual-level components**

- Providing a wearable device with real-time activity feedback (Fitbit Inspire HR; Google LLC)
- Using a Fitbit smartphone app
- Offering individual weekly lottery-based financial incentives, where 1 intervention group participant who participated in at least 70% of the movement breaks within the previous week was randomly selected to receive a reward of THB 500 (US $16)

**Social-level components**

- Team movement breaks of light to moderate intensity, lasting at least 4 minutes and occurring 4 times a day (alarm clocks with speakers were provided to movement break leaders to initiate sessions), which served as the primary intervention component
- Team-based incentives of an additional weekly lottery reward of THB 500 (US $16) given to the winner if at least 70% of the participants in the cluster also attended at least 70% of the movement breaks within the previous week; 4 alarm reminders were set (9:30 AM, 10:30 AM, 2:30 PM, and 3:30 PM), and trained movement break leaders managed the starting times, songs, and movements; participants working from home were encouraged to join the sessions via web conferencing

**Environmental-level component**

- Three types of posters providing information on health risks associated with high sedentary time and the benefits of physical activity, as well as examples of stretching exercises

**Organizational-level component**

- Leadership support in the form of messages sent by office directors twice a week via Line (LY Corporation) to encourage participants to reduce sedentary time using movement breaks and increase physical activity, as well as to announce reward winners with congratulatory messages and photographs of the reward ceremony

**Figure 1:**
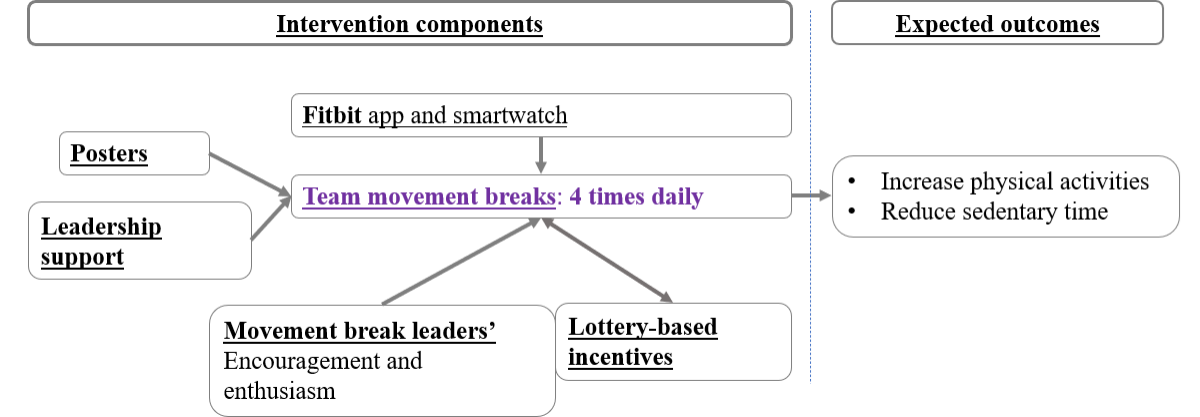
The mechanism of associations between the Physical Activity at Work cluster randomized trial intervention components and outcomes.

The multicomponent intervention was developed using the socioecological model [9,21]. The intervention components were strategically designed to complement one another, with a particular focus on the primary component: the movement breaks. In addition, department directors actively encouraged participants to attend more movement break sessions and announced the weekly lottery reward, which was contingent upon the frequency of participation in the movement breaks. Two movement break leaders per office were trained to oversee these sessions. Moreover, posters were displayed to inform participants about the adverse effects of prolonged sedentary behavior and to provide examples of strategies to break up sedentary time in the office. All intervention participants were equipped with a Fitbit Inspire HR smartwatch (Google LLC), preset to remind them to interrupt prolonged sitting every 30 minutes. Consequently, the intervention operated as illustrated in Figure 1.

We formulated our research questions to encompass the context, implementation, and impact mechanisms, with a specific emphasis on the primary intervention component: the movement breaks. This emphasis stemmed from two key considerations: (1) as previously noted, our intervention design places the movement break as the central intervention component, supported by other components; and (2) we observed a low participation rate (median 31.5%, IQR 20.4%-42.7%) in the movement break sessions. Moreover, 77.5% (107/138) of the intervention participants attended fewer than half of the breaks (240/480, 50%) throughout the intervention period. We hypothesized that the absence of a significant intervention effect could be attributed to low attendance in the movement break sessions [20]. Hence, our focus is to investigate the underlying reasons behind this issue. Table 1 describes the process evaluation components, specific questions, and the data sources.

**Table 1:** Descriptions of the Physical Activity at Work process evaluation components, specific questions, and the data sources.

Process evaluation components			Specific process evaluation questions		Quantitative data		Qualitative data
**Recruitment and context**							
	Recruitment	How does recruitment influence participation in movement breaks?		Recruitment rate		Recruitment fashion	
	Context	What impact do job descriptions and baseline characteristics of each cluster have on movement break participation?		Baseline summary statistics of each cluster		Job description of each cluster	
**Implementation**							
	Dose	What was the extent of intervention delivery?		Web monitoring: Fitbit wear time and conducts of movement breaks		On-site monitoring	
	Fidelity	Was the intervention delivered as intended?		Associations between participation in movement breaks and daily Fitbit sedentary time data as well as step counts during the intervention period		On-site monitoring	
**Mechanisms of impact**							
	Testing the assumptions behind the intervention design	How did supporting components influence participation in movement breaks?		Effects of intervention components on the percentage of weekly movement break participation		Focus group discussions	
	Participants’ attitude	What were the facilitators and barriers to participation in movement breaks?		Additional questionnaire regarding facilitators and barriers to participation in movement breaks		Focus group discussions	

### Ethical Considerations

Ethics approval for both the quantitative and qualitative components of this study was granted by the Ethical Review Committee for Research in Human Subjects, Ministry of Public Health, Thailand (IRB00001629), in accordance with relevant ethical guidelines for human research. Any modifications to the approved protocol will be submitted to the ethics committee for review. All participants provided written informed consent before participation, which included consent for the use of deidentified data in secondary analyses and for the publication of results. To protect participants’ privacy and confidentiality, all data were deidentified, and no personally identifiable information was included in the final dataset, with all published data presented in aggregate form. Participants’ data (eg, case record forms, laboratory test results, information sheets, and consent forms) are stored in a locked cabinet in a researcher’s office. All data will be destroyed by researchers within 5 years of publication. During the study, only deidentified data were used, and the data were only accessible to the research team. Participants received compensation of THB 250 (approximately US $7.5) for each data collection session in which they participated.

### Dissemination Policy and Authorship Guidelines

In addition to disseminating our research findings to the funder of this study, the Ministry of Public Health, we will disseminate our findings to other countries, the study participants, and the research community. We have followed the authorship guidelines of the International Committee of Medical Journal Editing.

### Data Collection

#### Baseline and Follow-Up Data Collection (Questionnaire)

An interviewer-administered questionnaire, based on the Thai National Statistical Office’s health survey and capturing sociodemographic data such as age and education [22], was used to collect participant data at both baseline and follow-up. In addition, intervention participants were asked supplementary questions regarding the implementation of the intervention during the follow-up data collection session (Multimedia Appendix 1).

#### Data Monitoring (Movement Break Schedule)

A data monitoring team, consisting of the trial implementer and an administrator, conducted weekly random field visits to 4 offices during the scheduled movement break sessions, adhering to the initial timetable. Monitored data included (1) the occurrence of sessions, (2) session quality, (3) participant attendance, and (4) any issues encountered during the session.

Movement break leaders were responsible for submitting weekly schedules via Microsoft Excel Online, indicating session timings and dates. Updates were required if a session did not fall within the 30-minute alarm windows (9:30 AM, 10:30 AM, 2:30 PM, and 3:30 PM), indicating absence or starting times outside the specified windows.

#### Fitbit Data and Movement Break Participation

During the 6-month intervention period, we collected Fitbit data, which included wear time, sedentary time, and step counts. Daily activity data were anonymized and used to compile weekly lists of potential reward recipients and to assess compliance with movement breaks. We determined participation in each movement break session by (1) analyzing a quadratic function of steps, (2) detecting quadratic functions within the 1-hour time frame of movement breaks, and (3) ensuring that participants had accumulated at least 100 steps.

### Quantitative Data Analysis

Linear mixed models were used to examine the associations between intervention components and activity outcomes, as well as between the intervention components themselves. These models incorporated random intercepts from both individual and cluster levels, while also accounting for random slope by including intervention weeks. Weekly individual data were derived from daily Fitbit records, follow-up questionnaire responses, and monitoring data (Table 2). Data were analyzed using RStudio (version 4.0.3; Posit Software, PBC) and Stata software (version 14.2; StataCorp LLC), with significance level set at 5%.

**Table 2:** Variables used in the Physical Activity at Work process evaluation.

Variable				Explanation		Scale
**Associations between movement break participation and daily Fitbit sedentary time and step count data during the intervention period**						
	**Outcome variables**					
		Sedentary time	Daily sedentary time data from Fitbit		Continuous (min)	
		Steps	Daily step count data from Fitbit		Continuous (step count)	
	**Exposure variables**					
		Movement break participation	Daily movement break participation		Ordinal; 1-4 (sessions)	
**Effects of intervention components on weekly movement break participation percentage**						
	**Outcome variable**					
		Movement break participation percentage	The frequency of an individual’s participation in movement break sessions within a specific week compared to the maximum number of sessions available during that week		Continuous (percentage)	
	**Exposure variables**					
		Individual reward	We designated the weeks following individual reward wins as {1}, while all other weeks were coded as {0}, using the web monitoring data		1=won a reward last week; 0=others	
		Cluster reward	We assigned {1} to the weeks following office colleagues’ reward wins and {0} to all other weeks, using the web monitoring data		1=a colleague in the same office cluster won a reward last week; 0=others	
		Fitbit wear time	We categorized the average weekly Fitbit wear time for each individual into a binary variable using the median		1=wore Fitbit at or above the median wear time; 0=wore Fitbit below the median wear time	
		Fitbit wear time (alternative)	How often Fitbit led individuals to engage in physical activity in the office in the last 2 weeks of the intervention		1=at least 4 d/wk; 0=<4 d/wk	
		Leadership support	How often the directors’ support led individuals to engage in physical activity in the office in the last 2 weeks of the intervention		1=at least 4 d/wk; 0=<4 d/wk	
		Movement break leaders	How the movement break leaders’ (1) encouragement and (2) enthusiasm contributed to movement break participation (We asked the movement break leaders and used the data as cluster-representative data)		3=both factors contributed *somewhat* or *a lot*; 2=only the encouragement contributed *somewhat* or *a lot*; 1=only the enthusiasm contributed *somewhat* or *a lot*; 0=both factors contributed *not at all*, *very little*, or *a little*	
		Posters	How often the posters led individuals to engage in physical activity in the office in the last 2 weeks of the intervention		1=at least 4 d/wk; 0=<4 d/wk	
		Posters (alternative)	How accurate individuals were in reporting the number of different poster designs in their office		1=3-4 designs; 0=0-2 designs	

### Focus Group Discussions

The qualitative aspect of the study involved focus group discussions aimed at exploring participants’ perspectives on facilitators and barriers to engagement in movement breaks, as well as their attitudes toward intervention components. Intervention clusters were initially ranked by mean percentage participation in movement breaks. Subsequently, using purposive sampling, we invited up to 6 participants with the highest percentage participation from the top 2 clusters and up to 6 participants with the lowest percentage participation from the bottom 2 clusters for each of the 4 focus group discussions.

The 4 focus group discussions took place via 45- to 75-minute Zoom (Zoom Video Communications, Inc) meetings, with participants joining from their respective offices. The focus group discussions were recorded with participants’ consent (both video and audio were captured), while notes were taken during the sessions. Before each focus group discussion, participants were briefed about its purpose, format, and estimated duration, with the option to interrupt as needed.

Verbatim transcriptions were manually completed by hired research assistants. The transcriptions were then subjected to deductive thematic analysis, with facilitator and barrier serving as overarching themes, and the socioecological model used as subtheme [9]. Two analysts (KA and WI) independently coded each transcript using the pre-established framework. References under themes and subthemes were compared and discussed to ensure consistency and accuracy. Intercoder reliability was not analyzed in this study.

An interview guide (Table S1 in Multimedia Appendix 2) was developed based on the research question “What are the facilitators and barriers to movement break participation?” The socioecological model informed this guide. A total of 4 interviewers (female: n=2, 50%) conducted the focus group discussions. They comprised the PAW trial implementer (first author; doctor of medicine), 2 study administrators (bachelor of communication arts and master of political science), and a research assistant (bachelor of clinical pharmacology) who was not a trial staff member. All interviewers underwent a comprehensive 2-day training program tailored to the qualitative data collection and analysis requirements of the study. This training was led by a Thai senior researcher (PhD in anthropology). The focus group discussion guide served as a reference tool, ensuring that critical topics were not overlooked. During the focus group discussions, the researcher flexibly adjusted the sequence, content, and style based on individual responses. Emotions and nonverbal cues expressed by participants were carefully documented.

Code names were used in place of real names in the recorded data to protect participant privacy. Data collection and analysis proceeded concurrently throughout the study. After coding the transcripts from the 4 focus group discussions, the researchers and supervisors deliberated on data saturation. Subsequently, 2 additional focus group discussions were conducted with participants from clusters ranked in the top and bottom thirds, following the same procedures. No new themes emerged from these final 2 focus group discussions, indicating that data saturation had been achieved.

## Results

### Recruitment and Context

The PAW study recruited participants from all clusters within the Department of Medical Services building and the International Health Policy Program, Ministry of Public Health, Thailand, between July and September 2020. The ministry’s governance structure is typically bureaucratic, with each department led by a single director general and each office headed by an office director. In total, 18 clusters were successfully recruited, with 15 (83%) falling under the Department of Medical Services purview; the remaining 3 (17%) clusters were under the jurisdiction of the Office of the Permanent Secretary (n=2, 67% were part of the International Health Policy Program and located in a distinct building complex; Figure S1 in Multimedia Appendix 2).

Recruitment procedures at the cluster level proceeded seamlessly. The directors of each office endorsed the active involvement of their respective teams in the trial, resulting in a cluster-level recruitment rate of 100% (18/18). For individual-level recruitment, we initiated the process by organizing office-specific group meetings to thoroughly explain the trial details. After these meetings, office workers interested in participating could engage with the trial staff to complete the individual informed consent process. To accommodate those unable to attend the scheduled meetings due to prior commitments, additional sessions were arranged.

Despite the bureaucratic governance style, the individual recruitment rate reached 62.9% (282/449). Job descriptions varied and included research-related roles and academic, finance, law, digital, and other administrative positions. At baseline, 3 (17%) of the 18 clusters exhibited a mean daily sedentary time exceeding 9 hours (mean sedentary time of 546, SD 90.3 min). Moreover, mean daily moderate to vigorous physical activity time was generally higher than the current physical activity guideline, reflecting that participants were relatively active. No significant associations were observed among job descriptions, baseline time spent in sedentary or moderate to vigorous physical activity, and monitoring data. However, clusters with higher baseline sedentary time demonstrated increased participation in movement breaks (Figure S2 in Multimedia Appendix 2). Furthermore, mean age and cluster size seemed to influence participation in movement breaks within the intervention group. Notably, individuals in the cluster with the youngest and smallest number of participants showed minimal participation in movement breaks (Table 3).

**Table 3:** Recruitment and context of intervention participants and the implementation of the Physical Activity at Work intervention (n=140).

	Baseline (n=140)				Data monitoring (6-mo intervention period) (n=138)			
Job description	Cluster size, n (%)	Recruitment rate n/N (%)	Sedentary time (min/d), mean (SD)	Lottery-incentive wins, n (%)		Movement break participation (%), mean (SD)	Maximum work from home, n/N (%)	Notes
Research related	23 (16.4	23/25 (92)	541 (90.5)	15 (63)		59.7 (14.7)	2.5/5 (50)	—^a^
Research related	8 (5.7)	8/44 (18)	483 (99.4)	0 (0)		25.8 (13.2)	5/5 (100)	Different building and department
Nursing	13 (9.3)	13/37 (35)	463 (117)	4 (17)		38.8 (16.6)	0/5 (0)	Different department
Finance	10 (7.1)	10/11 (90)	460 (95.9)	0 (0)		32.9 (5.49)	2/5 (40)	Broadcast from another cluster
Finance	14 (10.0)	14/17 (82)	481 (94.1)	1 (4.17)		29 (16.4)	2/5 (40)	—
Human resource	34 (24.3)	34/35 (97)	473 (113)	2 (8)		31.5 (11)	2.5/5 (50)	—
Human resource	18 (12.9)	18/31 (58)	394 (141)	1 (4)		25 (7.99)	2.5/5 (50)	Broadcast from another cluster
Digital	15 (10.7)	15/25 (60)	447 (116)	1 (4)		19.7 (9.83)	2/5 (40)	—
Inspection	5 (3.6)	5/18 (27)	350 (121)	0 (0)		3.88 (4.54)	2/5 (40)	n=3 (50%) participants at follow-up

^a^Not applicable.

### Implementation

#### Fidelity: Was the Intervention Delivered as Intended?

##### On-Site Monitoring

The on-site monitoring team conducted 65 field visits to the intervention group participants’ offices during their scheduled movement break sessions. During these visits, 35 movement breaks were observed, with 28 (80%) sessions featuring the Department of Medical Services theme song alongside another preferred Thai song. Importantly, there were no instances of cheating, such as merely shaking the Fitbit device without engaging in actual physical activity. However, 1 (8%) of the 13 participants from cluster 13 chose to take a 10-minute walk outside the office instead of participating in the team movement breaks, even when the sessions were not prompted. This participant managed to secure the weekly reward 3 times during the 24-week intervention period. Finally, no participants from the control group attended the sessions.

All alarm clocks were operational; nevertheless, leaders opted to broadcast the movement break songs using alternative devices, including the built-in broadcast system and office speakers. Notably, 2 (22%) of the 9 clusters did not independently initiate their movement break sessions; instead, they waited for another cluster to start the activity because they shared the same broadcast systems.

The movement break schedule reports submitted by the leaders were accurate; nonleaders who independently initiated sessions assisted with the reporting process. This collaboration ensured that the recorded data accurately reflected actual session initiation and participation. This also supports the finding that in some of the clusters (3/9, 33%), participants took the initiative to lead sessions without leader involvement, demonstrating shared responsibility in maintaining intervention fidelity.

Weekly rewards were distributed to winners by department directors. Participants received the team-based incentive on only 2 (8%) out of 24 occasions. A photograph capturing the reward ceremony, featuring face-to-face reward distribution, was shared with all intervention participants through Line OpenChat.

We received notifications regarding Fitbit issues, including syncing, freezing, and charging problems, both during and outside of our field visits. Fortunately, some participants with technological expertise were able to provide assistance, alleviating the need for the implementation team to respond to every notification. During our observations, we noticed that some of the participants (9/138, 6.5%) occasionally left their Fitbit devices at home when attending the movement break sessions. Despite understanding that their participation would not be recorded without the Fitbit, they still chose to engage in the sessions. In addition, 1 (0.7%) of the 138 participants was observed wearing the Fitbit only during the movement breaks.

All posters remained undamaged and visible, without any changes to their placement.

Due to the COVID-19 pandemic, a work-from-home policy was implemented. However, fewer than half of the participants (49/138, 35.5%) worked from home, with only 1 (11%) of the 9 clusters having a 100% work-from-home arrangement. This cluster used Zoom for meetings to conduct their movement breaks. Upon visiting the Zoom link twice, we found only 1 (0.7%) of the 138 participants in the Zoom meeting, with no movement breaks initiated. By contrast, another cluster coordinated their participation through a Line group chat when working from home, ensuring simultaneous session participation without requiring an online meeting.

##### Online Monitoring

We examined the correlations between engaging in movement breaks and daily Fitbit sedentary time and step count data. Each additional movement break was associated with a reduction of 6.20 (95% CI 6.99-5.41) minutes in sedentary time and an increase of 245 (95% CI 222-267) steps, adjusting for Fitbit wear time, number of public holidays in that week, and participants' age, sex, and education, with cluster and ID as random intercepts and intervention week number as a random slope.

This analysis, based on pedometer-measured outcomes, supported the fidelity of the movement break implementation because our prescribed minimum requirement for the duration of a single session was 4 minutes. Furthermore, the cumulative steps surpassed the eligibility criterion of 100 steps. The analysis also suggests successful data synthesis regarding movement break participation (Table 4).

**Table 4:** Associations between movement break participation on daily Fitbit sedentary time and step counts during the intervention period in the Physical Activity at Work cluster randomized trial.

	Model A			Model B	
	β^a^ (95% CI)	*P* value	β^b^ (95% CI)		*P* value
Sedentary time (min)	–6.32 (–7.06 to –5.57)	<.001	–6.20 (–6.99 to –5.41)		<.001
Steps (count)	263 (242 to 283)	<.001	245 (222 to 267)		<.001

^a^Linear mixed-effect model adjusted for Fitbit wear time, with cluster and ID as random intercepts and intervention week number as the random slope.

^b^Further adjusted for the number of public holidays in that week, age, gender, and education of the participants.

#### Dose: What Was the Extent of Intervention Delivery? (Online Monitoring)

According to the movement break leaders’ weekly reports, many movement break sessions were never initiated by the leaders in each cluster. By the third week of the intervention, approximately 40% (72/180) of the scheduled movement break sessions had not been initiated (Figure S3 in Multimedia Appendix 2). Similarly, the overall average participation in movement breaks declined after the third week of the intervention, reducing to an average of 8 sessions per week per participant. Subsequently, the participation rate continued to decrease (Figure S4 in Multimedia Appendix 2).

We plotted the average daily Fitbit wear time to assess adherence during the intervention period (Figure S5 in Multimedia Appendix 2). We found that participants typically wore the devices for 10 to 15 hours per day at the start, with a slight decrease to 8 to 14 hours occurring 2 to 3 months into the intervention.

### Mechanisms of Impact

#### Overview

Building on Figure 1, we present the effects of each component derived from a mixed methods analysis aimed at exploring the associations between each intervention component and movement break participation. We combine results from both quantitative and qualitative analyses to comprehensively address the question.

#### Quantitative Analysis

A total of 3200 participant-week data points were extracted by combining participants’ demographics, Fitbit wear time, sedentary time and step count data, answers from the attitude-toward-intervention-components questionnaire, and online monitoring of rewards (Table 2).

#### Qualitative Analysis

##### Overview

There were 28 participants (n=3, 11% to n=6, 21% in each session) across 6 focus group discussions. The primary data analysts for the main results included 4 interviewers: the head of the data collection team (male), a program administrator (female), a researcher (male), and a staff member from the Health Intervention and Technology Assessment Program (a semiautonomous research unit under the Ministry of Public Health; female). Demographic details of all participants from the clusters (coded aby C12, C13, C5, C8, C16, and C17) involved in the focus group discussions are presented in Table S2 in Multimedia Appendix 2. Generally, the mean age of participants from the 3 top-ranked clusters was higher than that of participants from the bottom-ranked clusters. The majority of the participants (≥80%; 23/28, 82%) were female. Notably, the cluster with the lowest participation rate of 2.1% (10/480) comprised only 3 (2.2%) of the 138 participants at the 6-month follow-up (Table S2 in Multimedia Appendix 2).

##### How Did the Supporting Components Influence Participation in Movement Breaks?

#### Lottery-Based Incentives

Individuals who won the weekly lottery rewards the previous week demonstrated an 8.64% (95% CI 0.985%-16.3%) increase in movement break participation compared to other data points (Table 5). However, it is crucial to note the possibility of an overestimation when comparing data from winners against those from nonwinners. To address this, we conducted another subgroup analysis exclusively including winners, revealing a 5.1% (95% CI –3.44% to –13.6%) increase in movement break participation. However, this increase lacked statistical significance due to the small sample size (Table S3 in Multimedia Appendix 2).

**Table 5:** Effects of the Physical Activity at Work intervention components on weekly movement break participation percentage.

			β^a^ (%; 95% CI)		Standardized β (%; 95% CI)		*P* value
Individual reward			8.64 (.985 to 16.3)		.0257 (.00293 to .0484)		.03
Cluster reward			–.325 (–2.64 to 1.99)		–.004 (–.0325 to .0245)		.78
Fitbit wear time			3.96 (2.28 to 5.65)		.0696 (.04 to .0993)		.001
**Leadership support**			1.82 (–2.25 to 5.9)		.0309 (–.0381 to .1)		.38
	Enthusiastic	2 (–17.2 to 21.2)		0.077 (–.663 to .817)		.84	
	Encouraging	1.49 (–18.8 to 21.8)		0.0576 (–.724 to .84)		.89	
	Both enthusiastic and encouraging	24.1 (8.96 to 39.2)		0.929 (.346 to 1.51)		.002	
Posters			4.49 (–.493 to 9.47)		0.0695 (–.00764 to .147)		.08

^a^Linear mixed effects model adjusted for the previous-week movement break participation percentage, number of public holidays in that week, age, sex, and education of the participants, with cluster and ID as random intercepts and intervention week number as the random slope.

During the focus group discussions, some of the participants suggested that rather than a single winner receiving a substantial cash prize, there should be multiple winners receiving more affordable rewards:

Rather than cash, it should be acknowledgments, such as showing who reach this many steps so we can compete with each other.

C13

[C]ould be something cheaper, don’t have to be cash. Cheap shirts will do!

C10

[I]t was indeed a motivating factor, but the conditions for obtaining it should be somewhat more lenient. That is, to make it accessible to a broader audience, but the current conditions seem to be quite high. If it were reduced a bit, say to 100, I believe that providing rewards would be motivating enough.

C8

A participant in the worst-performing cluster did not remember the details of the financial incentive:

Sorry but how much was the reward?

C17

#### Team-Based Incentives

We observed no difference in movement break participation percentage (–0.325%, 95% CI –2.64% to 1.99%) in the weeks after a colleague from the same office won lottery rewards compared to other weeks. Contrary to our assumptions during the intervention development phase, peer pressure seems to have had no discernible effects on motivating movement break participation, as indicated by the quantitative analysis model (Table 5).

Different ideas emerged during the focus group discussions, as outlined in the following paragraphs.

Peer support was mentioned among members in the best-performing cluster as an important motivator:

Because it helped the team. If we dance, someone gets 1000THB, if we don’t, it’s 500THB only.

C12

However, others expressed discouragement:

When I see others got it and I never won for weeks, I was disheartened.

C12

Some of the participants viewed rewards as not motivating:

I joined the sessions because I want to be healthier. Rewards are meaningless for me.

C13

#### Fitbit Wear Time

There was a 3.96% (95% CI 2.28%-5.65%) increase in movement break participation among individuals with higher Fitbit wear time compared to those with lower wear time, using the median weekly wear time as the cutoff (Table 5). However, this increase may be attributed to motivation triggered by Fitbit notifications (eg, reminders to break prolonged sitting) or potentially to an information bias, wherein participants who wore their Fitbit devices for longer periods were detected more frequently in movement break participation.

To address potential bias, we compared the result with another model where Fitbit exposure was based on self-report, using the question “How often did you look at the Fitbit tracker during the last 2 weeks of the intervention period?” The analysis revealed that looking at the tracker more frequently (≥4 times/wk) was associated with a 1.97% (95% CI –1.42% to 5.36%) higher movement break participation percentage, without statistical significance (Table S4 in Multimedia Appendix 2).

Qualitative analysis indicated that participants perceived Fitbit as beneficial not necessarily for encouraging additional movement breaks but rather for motivating increased exercise and providing real-time feedback:

I joined short breaks a lot at first, but after a while, I forgot...However, I always wear the watch; look at the daily data.

C13

I set my goal to walk 10,000 steps a day after I got the watch, and I succeeded!

C16

[S]aying Fitbit encourages more movement breaks is wrong for me, but I feel the urge to exercise more from wearing the watch, like running after work.

C17

#### Leadership Support

Participants perceived the encouragement from directors as ineffective in motivating them to participate in more movement breaks.

Although office directors joined very few movement breaks, participants believed that their presence helped motivate everyone in the cluster:

He joined, I saw. But mostly he’s busy.

C16

It made us stand up and dance...if we didn’t it’d be awkward.

C5

We all danced every time our director was present (laugh).

C8

With regard to the movement break leaders’ encouragement and enthusiasm, clusters with leaders who self-evaluated as more encouraging and enthusiastic had a significant increase of 24.1% (95% CI 8.88%-39.4%) in movement break participation compared to clusters with less encouraging and less enthusiastic leaders. However, because these exposure variables are self-reported by cluster leaders, the detected difference may be influenced by other cluster-specific factors.

Participants believed that movement break leaders’ enthusiasm and encouragement helped them join more break sessions:

He was very active and always encourages everyone to stand up and dance.

C8

We were aware of the scheduled time, but if we were occupied, the leader would notify us.

C13

Nevertheless, some of the participants mentioned that they did not rely on leaders:

No matter how many people in the office, we danced. no leaders, no problem at all.

C12

I’m not sure...leaders always initiated the activity, but we did not always join.

C16

No one was available.

C17

#### Posters

Participants who reported that posters motivated them to engage in more movement breaks during the last 2 weeks of the intervention exhibited approximately a 5% higher participation rate, although this increase lacked statistical significance (Table 5). Notably, this increase may indicate participants’ attitudes toward the intervention component rather than the direct effects of the posters themselves. Therefore, we compared the result by converting the variable to whether participants accurately identified the number of different styles of posters in their offices. The analysis revealed no significant difference in movement break adherence between those who answered correctly and those who did not (standardized β=1.94, 95% CI –1.34 to 5.23; Table S5 in Multimedia Appendix 2).

Qualitative analysis showed that while the posters initially captured interest, over time, they failed to sustain attention:

At first I read them. I thought it was helpful and tried to follow some moves. After a while I just ignored them.

C2

I didn’t really read it that much...just walk past.

C3

The standardized β coefficients of all exposures indicate that the self-evaluated enthusiasm and encouragement of movement break leaders were the most important variables among those included, followed by Fitbit wear time and the individual reward (Table 5).

##### Facilitators and Barriers to Movement Break Participation

**Figure 2:**
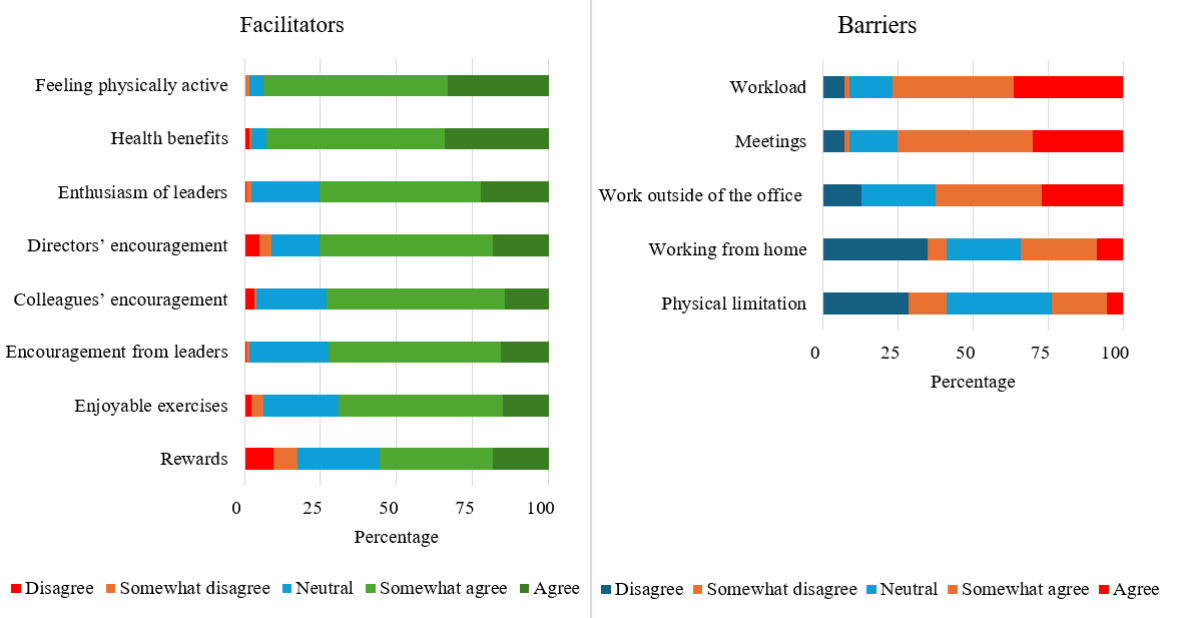
Facilitators and barriers to movement break participation in the Physical Activity at Work cluster randomized trial.

Figure 2 illustrates participants’ attitudes regarding the facilitators and barriers to movement break participation. The top 2 facilitators were the positive feelings associated with being physically active and the perceived health benefits, followed by encouragement from leaders, directors, and colleagues. The appeal of engaging in enjoyable exercises also motivated their participation. Interestingly, weekly rewards ranked lowest among the facilitators.

Conversely, the primary barrier perceived by participants was their workload, followed by meetings and working outside of the office. Surprisingly, many participants did not view working from home as a barrier to their participation.

Thematic analysis revealed positive attitudes toward intervention components:

I think having Fitbit is a really good motivation to move.

C17

Previously when someone invited me to run, I wanted to but it was hard. This [movement breaks] is easier and fun.

C12

Participants also noted barriers to the intervention, such as the lottery reward design:

It [individual reward] somehow motivated me, but I guess I just could not do as good as others.

C16

Another important barrier was the monotonous design of the movement breaks:

Dancing with the same moves gets boring after a while.

C8

I think we can change the songs to make it more interesting.

C16

Table 6 presents thematic analysis results with references under each theme and subtheme from all 6 focus group discussions.

**Table 6:** Thematic analysis of focus group discussions on the facilitators and barriers to movement break participation in the Physical Activity at Work cluster randomized trial.

Themes and subthemes		Quotes
**Facilitators**		
	Psychological capability	“Previously when someone invited me to run, I wanted to, but it was hard. This is easier and fun.” (C12)
	Social opportunity	“Both songs and moves...it was like mass hysteria; others enjoyed the sessions and I wanted to join” (C12) “Our director joined, and we danced happily” (C8) “When my boss dance and I just sat there working, it felt strange, so I joined” (C5)
	Physical opportunity	“I’m happy that there is a new opportunity for me to exercise...in workplace. Normally I don’t have time.” (C12) “Our office is spacious. There are empty spaces for dancing.” (C5)
	Reflective motivation	“My personal goal was actually health, not rewards” (C13) “I set my goal to get the reward and lose weight and cholesterol level because the programme measured those” (C5)
	Automatic Motivation	“It was relaxing, both mind and body...especially the mind.” (C17) “Just that we got to dance...when the session started, it relieved the stress quite a lot” (C5)
**Barriers**		
	Psychological capability	“10 min. dancing is too short. 30 min. working out is better for your health for the day.” (C13) “My programming work never allows me to lose focus” (C16)
	Social opportunity	“Meetings definitely prevented us to join movement sessions” (C8) “We were not really motivated by directors and colleagues” (C17)
	Physical opportunity	“Sometimes workspace is not wide enough...also I was afraid I'll annoy others who were not in the project” (C13) “During high workload, we cannot join” (C5) “The high workload never allows us to do anything else” (C17) “Ever since COVID situation got worse, I’ve been sitting all the time at my desk. It’s the workload, can’t do anything else.” (C16)
	Reflective motivation	“Rewards could be anything, like shoes, incentives don’t need to be money, like shirts...” (C5)
	Automatic motivation	“Each programme shouldn’t last long. I mean, we should always change the stimulant to avoid boredom...” (C13) “Dancing with the same moves gets boring after a while” (C8) “I think we can change the songs to make it more interesting.” (C16)

## Discussion

The PAW cluster randomized trial of a multicomponent intervention, developed based on the socioecological framework [9], showed a decrease in participants’ waking sedentary time and an increase in moderate to vigorous physical activity, although without statistical significance [20]. We conducted a mixed methods process evaluation, following the Medical Research Council’s guidance [11], to comprehensively describe the PAW trial’s (1) recruitment and context, (2) implementation, and (3) impact mechanisms.

### Recruitment and Context

Our team anticipated a high recruitment rate once the office directors approved the inclusion of their offices in the trial, given the bureaucratic nature of the ministry organization in Thailand. However, recruitment rates were low in many clusters, weakening the trial’s statistical power and risking reduced intervention adherence, particularly in the social-level component.

Cluster 17 serves as a notable example, demonstrating minimal engagement in movement breaks (Table 3; Figure S4 in Multimedia Appendix 2). This could be attributed to the recruitment of too few, relatively young participants alongside a high number of nonparticipants (Tables 3 and 4). Despite its potential for easy qualification for the team-based incentive with just 3 participants (at follow-up), cluster 17 might have faced challenges due to the negative influence of an environment where breaks are discouraged. This aligns with a previous study reporting reduced break taking in disapproving work environments compared to more supportive ones [23]. Cluster 13 also faced challenges with a low recruitment rate; yet, the engagement of the participants in this cluster in movement breaks was high. Two reasons might explain this: (1) the participant who chose to walk outside the office and secured 3 weekly rewards increased the cluster’s average participation rate, and (2) recruiting at least 13 participants could create an environment conducive to making movement breaks feel enjoyable and secure to participate in.

Job characteristics might also play a role in movement break participation. Cluster 17, “the inspection office,” frequently required its members to leave the office for inspections, which was reflected in their low sedentary time at baseline (Table 3) and echoed in the focus group interviews. In addition, cluster 16 represented a digital office where most participants worked on their laptop computers all day. While this setting might seem ideal for implementing the intervention, participants encountered challenges with breaks because they needed continual focus to code*.* This aligns with the suggestion that identifying the optimal fit between the organizational context and the intervention is crucial [24]. However, challenges remain when implementing such interventions across organizations with diverse offices, each characterized by unique job contexts and work styles.

### Implementation

We implemented the trial with a comprehensive dose delivery, ensuring intervention fidelity by closely adhering to the original plan for each component. Moreover, the findings in Table 3 affirm the application of precise criteria for enumerating movement breaks, underscoring their correlations with the expected reductions in sedentary time and increases in step counts. Nevertheless, we encountered challenges related to the frequency of movement break sessions conducted by participants, which we refer to as “dose received” [13]. This inconsistency may be attributed to the performance of the movement break leaders, as illustrated in Figure S3 in Multimedia Appendix 2, which shows that many sessions were never initiated. However, several clusters relied on automatic timing mechanisms to initiate breaks, ensuring that sessions commenced even without leaders present. We discuss this further in the next subsection.

### Mechanisms of Impact

#### Testing the Designed Intervention Components

Healthy behaviors are maximized when environments and policies support healthful choices, and individuals are motivated and educated to make them [25]. As one of the main principles of the ecological model, the interaction of influences means that variables within the system work together synergistically [26]. Hence, our study examined the impact of the supporting components on the movement break participation using multivariate linear mixed models (Table 4).

The enthusiasm and encouragement of the movement break leaders seem to have contributed to higher participation percentages. However, the cluster that exhibited the best performance challenged this observation, asserting that the members initiated sessions independently, even without leaders. Unlike other cluster randomized trials where exercise sessions were led by nonparticipants such as physiotherapists [16], the movement break leaders in our study were participants and could be replaced if absent. Hence, without the leaders, other members could initiate sessions automatically. Nevertheless, the influence of the leaders’ encouragement and enthusiasm likely played a role in motivating the rest of the team. Prior research has also found that workplace team leaders play a significant role in facilitating the implementation of workplace interventions [27,28]. Moreover, a comprehensive review emphasizes that effective team performance underscores the fulfillment of leadership styles, supportive team behaviors, communication, and performance feedback [29].

The impact of the team-based incentive on movement break participation was evidently negligible. Regarding individual lottery-based incentives, their influence remains somewhat unclear. Systematic reviews suggest that financial rewards for physical activity have positive short-term effects, surpassing unconditional incentives [30,31]. Moreover, another study indicates that increasing reward values may lead to improved results [32]. Nevertheless, insights from online monitoring data and focus group discussions revealed that only 1 (11%) of the 9 clusters actively pursued rewards and engaged in friendly competition, while the others (8/9, 89%) were indifferent. Awarding only 1 winner per week might have demotivated participants who faithfully adhered to the intervention but never won. This phenomenon can be explained using goal-setting theory, which suggests that setting clear and appropriately challenging goals is crucial [33]. By contrast, setting unachievable goals may induce stress, anxiety, and perceived pressure [34]. Therefore, overly difficult goals, such as securing the weekly lottery reward, may have discouraged participants.

Fitbit was perceived as a helpful tool for real-time data monitoring and might help motivate leisure physical activity. However, current evidence indicates unfavorable outcomes regarding the effectiveness of pedometers in increasing physical activity within the workplace or in motivating sedentary breaks [35,36]. The component may have primarily served as a data collection tool rather than actively supporting movement breaks. By contrast, the posters, which served as the environmental component, proved ineffective and should be replaced with physical environment interventions, which have been shown to be more effective [4,8].

Finally, leadership support was considered helpful at the start of the intervention but provided no lasting effect over time. The component could be perceived as a nudge, which has been widely used to prompt behavioral change among participants by providing alternative options to sedentary habits in the workplace [37]. Leadership support was expected to facilitate these behavioral shifts. Management support has also been found to be an essential enabler for workplace intervention participation to reduce sitting time [27,38,39]. However, the sustainability of the effects depends on the meticulous design of the intervention component. Future studies evaluating long-term effects must enhance the intervention component to ensure sustainability.

#### Facilitators and Barriers

Automatic motivation, driven by the desire to feel active and relaxed, was ranked as the top facilitator of movement break participation, followed by reflective motivation, including perceived health benefits. The findings are in line with a recent systematic review [39]. Another review also supports the idea that microbreaks at work can improve well-being by boosting vigor and reducing fatigue [40]. In addition, participants generally grasped the concept of health benefits associated with movement breaks. However, some of the individuals compared movement breaks to aerobic exercise, expressing doubt regarding their health benefits*.*

Workload and meetings were perceived as the main barriers to movement breaks, which aligns with a previous study’s prediction that high workloads, although positively related to the desire to detach from work, would also deter employees from actually taking breaks [23]. In addition, recent findings from a systematic review supported the notion that microbreaks increase well-being but do not necessarily improve work performance, especially in tasks that require high cognitive engagement. Moreover, the review suggested that breaks of >10 minutes may be necessary to enhance work performance [40].

By contrast, meetings presented clear barriers to our intervention design. Future research should be dedicated to advocating feasible and context-specific active meetings within an active workplace; for example, an exploratory study found that standing meetings were feasible, well-received by employees, and may reduce sitting time among the population [41]. However, widespread adoption faces obstacles due to prevailing sedentary work cultures and concerns about self-consciousness in front of senior staff, highlighting the need for broader social behavior change initiatives [42].

We hypothesized that movement break participation might be low due to the work arrangement during the peaks of the COVID-19 pandemic [20]. However, participants did not consider working from home to be the main barrier to movement break participation. Instead, they thought that the COVID-19 pandemic did not significantly hinder their adherence to the intervention due to the work-from-home policy. Instead, they attributed the difficulty to increased workload. Nevertheless, working from home remained 1 of the barriers to movement break participation. This is in line with another workplace cluster randomized trial, which reported that engaging in physical exercise with colleagues during working hours was more effective than home-based exercise in enhancing vitality and managing pain-related concerns among health care workers [43].

### Strengths and Limitations

We conducted a comprehensive mixed methods process evaluation of the PAW multicomponent intervention, covering recruitment and context, implementation, and impact mechanisms. Rigorous analyses were applied to both quantitative and qualitative data. The data are prospective, spanning 6 months of follow-up. Although the overall results show no significant impact of the intervention, this process evaluation offers insights that could be crucial for the future development and evaluation of intervention packages aimed at reducing sedentary time while improving physical activity levels.

Nevertheless, our study had some limitations. First, we could not test mediators to understand the underlying mechanisms through which one variable influences another due to the trial’s lack of efficacy [20]. Second, constructing intervention theories will involve reconstructing a logic model [11,44], a task we plan to undertake in future studies. Third, we could not complete the management and analysis of process evaluation data before the conclusion of the PAW trial. As a result, the process evaluation was conducted post hoc to elucidate the trial outcomes. Furthermore, our evaluation only incorporated participants’ data and perspectives, overlooking input from other stakeholders such as organizational directors and nonparticipants. Finally, the team members responsible for process evaluation were also involved in the outcome evaluation. While our team members possess the most comprehensive understanding of the trial details, potential bias in interpretations must be acknowledged [11].

### Conclusions

The PAW trial did not significantly reduce sedentary time among Thai office workers. Although the trial implementation was satisfactory regarding dose delivery and fidelity, there was limited uptake of the movement breaks, the key intervention component. This limited uptake could be attributed to (1) context-related challenges (including jobs requiring high cognitive engagement or frequent out-of-office work and meetings), (2) the absence of goal-setting aspects in the detailed design of individual and social components, (3) the lack of effective and sustainable supporting components at the environmental and organizational levels, and (4) elevated workloads in specific clusters (exacerbated during peak periods of the COVID-19 pandemic) serving as a significant barrier.

## Appendix

Multimedia Appendix 1 Additional questions for intervention participants.

Multimedia Appendix 2 Supplementary figures and tables.

## Data Availability

The datasets generated and analyzed during this study are available from the corresponding author on reasonable request.
