# Supplementary material for: The Physical Activity at Work (PAW) Program in Thai Office Workers: Mixed Methods Process Evaluation Study
Source: JMIR Form Res. 2025 Jan 2;9:e57604. doi: 10.2196/57604 (PMC11739726; doi:10.2196/57604)
Supplement: Multimedia Appendix 1 [file formative_v9i1e57604_app1.docx]

**Figure S1.** The simplified map of the Physical Activity at Work cluster-randomised trial sites


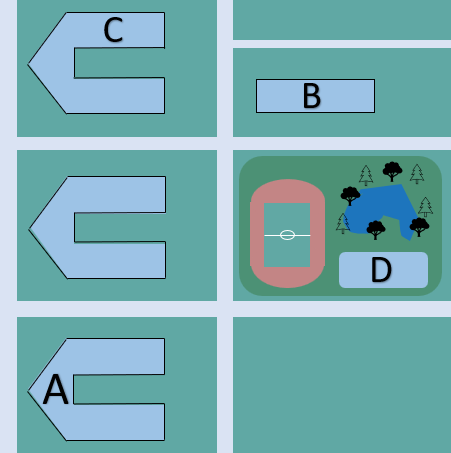


A: Department of Medical Services buildings (16 recruited offices)

B: International Health Policy Program building (2 recruited offices)

C: Health Intervention and Technology Assessment Program building (Implementors’ office)

D: Ministry of Public Health Sport Complex

**Figure S2.** Scatter plot of clusters’ mean baseline sedentary time and movement break participation in the Physical Activity at Work cluster-randomised trial

**Figure S3.** Movement break champions report of missing movement break conducts across all clusters in the Physical Activity at Work cluster-randomised trial

**Figure S4.** Daily movement break participation by cluster in the Physical Activity at Work cluster-randomised trial


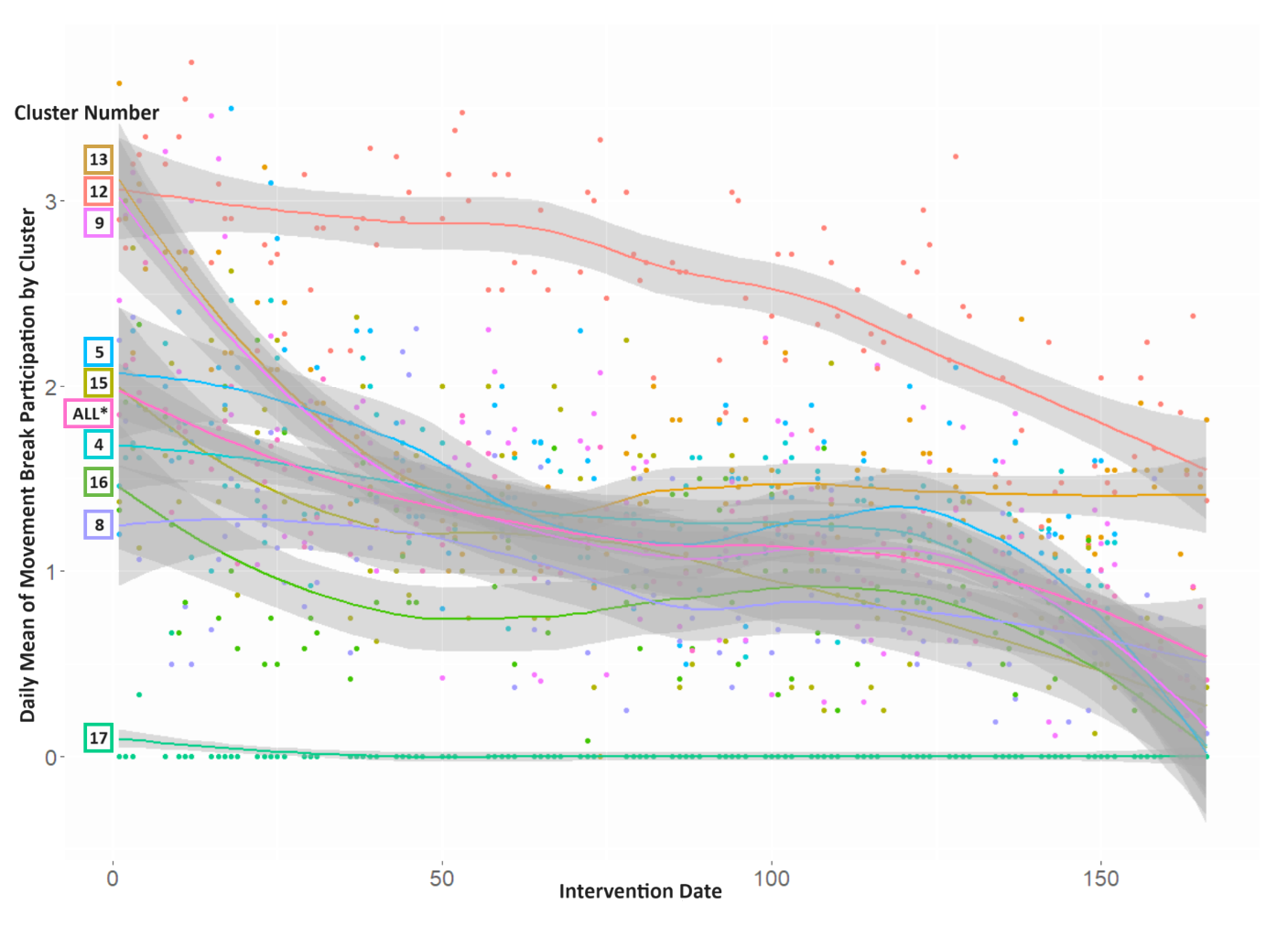


*ALL*: all participants’ data*

**Figure S5.** Average daily Fitbit® wear time (hours) among all intervention participants in the Physical Activity at Work cluster-randomised trial
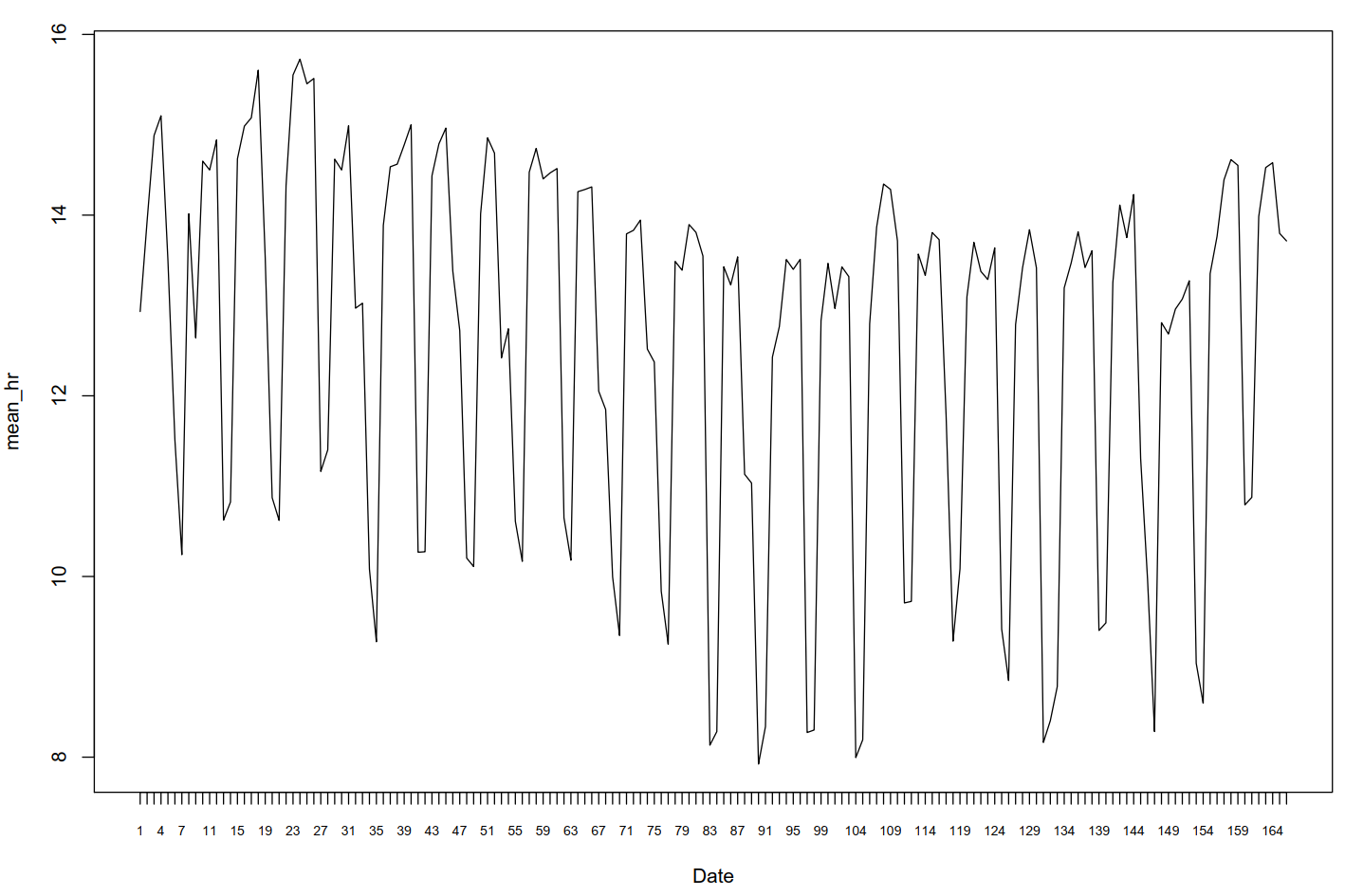


| **Table S1.** Topic Guide used in the focus-group discussion of the Physical Activity at Work process evaluation |
| --- |
| How did overall intervention encourage you? |
| What helped/hindered you the most? |
| How did Fitbit help? |
| What was your goal regarding lottery reward? |
| What were your (other) individual goals? |
| Any subjective drive? |
| How were the activity leaders? |
| Were there peer pressure? How? |
| How did work culture effect you? |
| How did director's encouragement help? |
| Explain how your job helped/hindered? |
| What did you get from the posters? |
| What about other environmental factors? |

| **Table S2.** Demographics of clusters included in focus group discussions of the Physical Activity at Work process evaluation | | | | | |  |
| --- | --- | --- | --- | --- | --- | --- |
| Demographics\Cluster | 12​ | 13 | 5​ | 8 | 16​ | 17 |
| No. of participants  - in the clusters | 21 | 12 | 10 | 16 | 12 | 3 |
| - in the focus group discussions | 6 | 5 | 5 | 4 | 5 | 3 |
| Age, mean (SD) | 42.2 (10.5) | 48.0 (9.31) | 44.8 (8.64) | 34.4 (7.68) | 39.6 (8.96) | 30.3 (5.03) |
| Female, n (%) | 17 (81) | 12 (100) | 7 (70) | 11 (69) | 9 (75) | 3 (100) |
| Percent participation in movement breaks​, mean (SD) | 59.7 (14.7)​ | 38.8 (16.6) | 32.9 (5.49) | 25.0 (7.99) | 19.7 (9.83) | 3.88 (4.54) |

| **Table S3.** Subgroup analysis of the impact of intervention components on weekly movement break participation percentage among movement breaks winners in the Physical Activity at Work cluster-randomised trial | | | |
| --- | --- | --- | --- |
|  | Beta ᵃ | Standardised Beta | p-value |
| Individual reward | 5.10  (-3.44 – 13.6) | 0.0426  (-0.0288 – 0.114) | .242 |
| Cluster reward | 0.588  (-4.49 – 5.66) | 0.00972  (-0.0741 – 0.0936) | .82 |
| Fitbit wear time | -22.7**  (-36.4 – -8.97) | -0.382**  (-0.613 – -0.151) | .001 |
| Leadership support | 16.9**  (6.81 – 27.0) | 0.272**  (0.110 – 0.435) | .001 |
| Movement break leaders’ encouragement and enthusiasm | 20.1**  (39.1 – 1.02) | 0.524**  (1.02 – 0.0267) | .039 |
| Posters | 2.87  (-7.53 – 13.3) | 0.0429  (-0.113 – 0.199) | .589 |
| ᵃ linear mixed-effect model adjusted for the previous-week movement break participation percentage, number of public holidays in that week, age, gender, and education of the participants, with cluster and ID as random intercepts and intervention week number as the random slope  **p < 0.05 | | | |

| **Table S4.** Impact of intervention components on weekly movement break participation percentage in the Physical Activity at Work cluster-randomised trial (converting the Fitbit® wear time into subjective reporting of checking data on Fitbit® more) | | | |
| --- | --- | --- | --- |
|  | Beta ᵃ | Standardised Beta | p-value |
| Individual reward | 8.41**  (0.719 – 16.1) | 0.0250**  (0.00214 – 0.0478) | .032 |
| Cluster reward | -0.386  (-2.72 – 1.95) | -0.00475  (-0.0334 – 0.0239) | .746 |
| Fitbit checking | 1.97  (-1.42 – 5.36) | 0.0333  (-0.0241 – 0.0907) | .256 |
| Leadership support | 1.54  (-2.65 – 5.73) | 0.0262  (-0.0449 – 0.0972) | .47 |
| Movement break leaders |  |  |  |
| - Enthusiastic | 2.90  (-16.4 – 22.2) | 0.112 (-0.633 – 0.857) | .769 |
| - Encouraging | 1.72  (-18.7 – 22.2) | 0.0665  (-0.721 – 0.854) | .869 |
| - both Enthusiastic and Encouraging | 24.5**  (9.31 – 39.8) | 0.380**  (0.144 – 0.616) | .002 |
| Posters | 4.83  (-0.319 – 9.99) | 0.074  (-0.00495 – 0.155) | .066 |
| ᵃ linear mixed-effect model adjusted for the previous-week movement break participation percentage, number of public holidays in that week, age, gender, and education of the participants, with cluster and ID as random intercepts and intervention week number as the random slope  **p < 0.05 | | | |

| **Table S5.** Impact of intervention components on weekly movement break participation percentage in the Physical Activity at Work cluster-randomised trial (converting the poster variable into accurately recalling the number of poster styles) | | | |
| --- | --- | --- | --- |
|  | Beta ᵃ | Standardised Beta | p-value |
| Individual reward | 8.76**  (1.09 – 16.4) | 0.0260  (0.00325 – 0.0488) | .025 |
| Cluster reward | -0.397  (-2.72 – 1.93) | -0.00489  (-0.0335 – 0.0237) | .737 |
| Fitbit wear time | 4.04**  (2.34 – 5.74) | 0.0684  (0.0396 – 0.0972) | < .001 |
| Leadership support | 4.13**  (0.613 – 7.64) | 0.0700  (0.0104 – 0.130) | .021 |
| Movement break leaders |  |  |  |
| - Enthusiastic | 2.44  (-16.3 – 21.2) | 0.0417  (-0.278 – 0.362) | .798 |
| - Encouraging | 2.30  (-17.6 – 22.2) | 0.0392  (-0.300 – 378) | .821 |
| - both Enthusiastic and Encouraging | 24.7**  (9.88 – 39.4) | 0.421  (0.169 – 0.673) | .001 |
| Posters | 4.83  (-0.319 – 9.99) | 1.94  (-1.34 – 5.23) | .246 |
| ᵃ linear mixed-effect model adjusted for the previous-week movement break participation percentage, number of public holidays in that week, age, gender, and education of the participants, with cluster and ID as random intercepts and intervention week number as the random slope  **p < 0.05 | | | |
