# Supplementary material for: The Physical Activity at Work (PAW) Program in Thai Office Workers: Mixed Methods Process Evaluation Study
Source: JMIR Form Res. 2025 Jan 2;9:e57604. doi: 10.2196/57604 (PMC11739726; doi:10.2196/57604)
Supplement: Multimedia Appendix 2 [file formative_v9i1e57604_app2.docx]

Additional File 1: additional questions for intervention participants

| **Description** | **Type** | **Possible values** |
| --- | --- | --- |
| How many types of PAW posters are there in the office? | numeric | 0, 1, 2, 3, 4 |
| factor contributed to you joining the movement breaks: Health | numeric | 1 Not at all 2 Very little 3 A little 4 Somewhat 5 A lot |
| factor contributed to you joining the movement breaks: Feeling physically or mentally good after exercising | numeric | 1 Not at all 2 Very little 3 A little 4 Somewhat 5 A lot |
| factor contributed to you joining the movement breaks: Rewards | numeric | 1 Not at all 2 Very little 3 A little 4 Somewhat 5 A lot |
| factor contributed to you joining the movement breaks: Colleagues’ encouragement | numeric | 1 Not at all 2 Very little 3 A little 4 Somewhat 5 A lot |
| factor contributed to you joining the movement breaks: Bosses’ encouragement | numeric | 1 Not at all 2 Very little 3 A little 4 Somewhat 5 A lot |
| factor contributed to you joining the movement breaks: The nice exercises | numeric | 1 Not at all 2 Very little 3 A little 4 Somewhat 5 A lot |
| factor contributed to you joining the movement breaks: Encouragement from movement break champion | numeric | 1 Not at all 2 Very little 3 A little 4 Somewhat 5 A lot |
| factor contributed to you joining the movement breaks: Enthusiasm of movement break champion | numeric | 1 Not at all 2 Very little 3 A little 4 Somewhat 5 A lot |
| factor contributed to you joining the movement breaks: Working from home | numeric | 1 Not at all 2 Very little 3 A little 4 Somewhat 5 A lot |
| factor reduced the number of times that you joined the movement breaks: Workload | numeric | 1 Not at all 2 Very little 3 A little 4 Somewhat 5 A lot |
| factor reduced the number of times that you joined the movement breaks: Working outside of the office | numeric | 1 Not at all 2 Very little 3 A little 4 Somewhat 5 A lot |
| factor reduced the number of times that you joined the movement breaks: Meetings | numeric | 1 Not at all 2 Very little 3 A little 4 Somewhat 5 A lot |
| factor reduced the number of times that you joined the movement breaks: Physical pain or injury | numeric | 1 Not at all 2 Very little 3 A little 4 Somewhat 5 A lot |
| factor reduced the number of times that you joined the movement breaks: Working from home | numeric | 1 Not at all 2 Very little 3 A little 4 Somewhat 5 A lot |
| How often did the following components lead you to engage in physical activity in the office in the last two weeks of the intervention?: Posters | text | 0 Never 1 1 day per week 2-3 2-3 days per week 4 4 days per week 5 5 days per week or more |
| How often did the following components lead you to engage in physical activity in the office in the last two weeks of the intervention?: Leader support | text | 0 Never 1 1 day per week 2-3 2-3 days per week 4 4 days per week 5 5 days per week or more |
| How often did the following components lead you to engage in physical activity in the office in the last two weeks of the intervention?: Movement break champion | text | 0 Never 1 1 day per week 2-3 2-3 days per week 4 4 days per week 5 5 days per week or more |
| How often did the following components lead you to engage in physical activity in the office in the last two weeks of the intervention?: Movement break reward | text | 0 Never 1 1 day per week 2-3 2-3 days per week 4 4 days per week 5 5 days per week or more |
| How often did the following components lead you to engage in physical activity in the office in the last two weeks of the intervention?: Fitbit app and watch | text | 0 Never 1 1 day per week 2-3 2-3 days per week 4 4 days per week 5 5 days per week or more |
